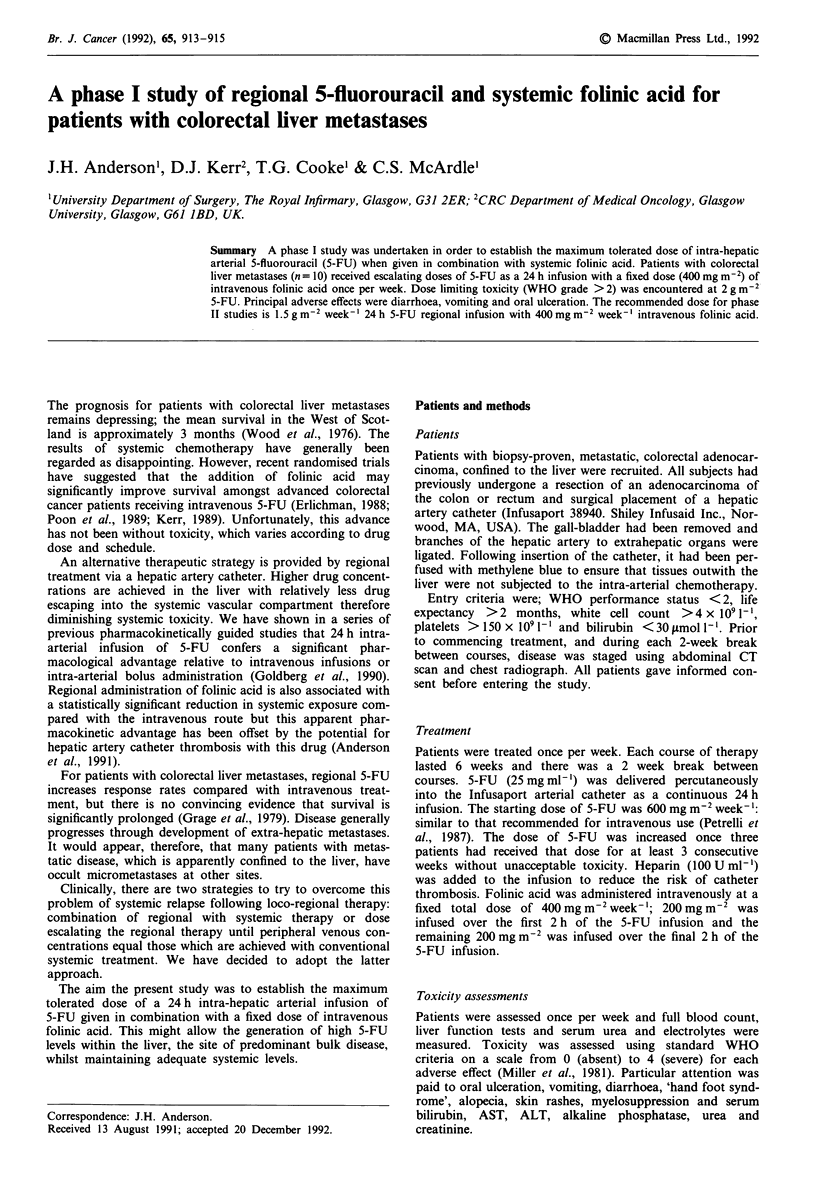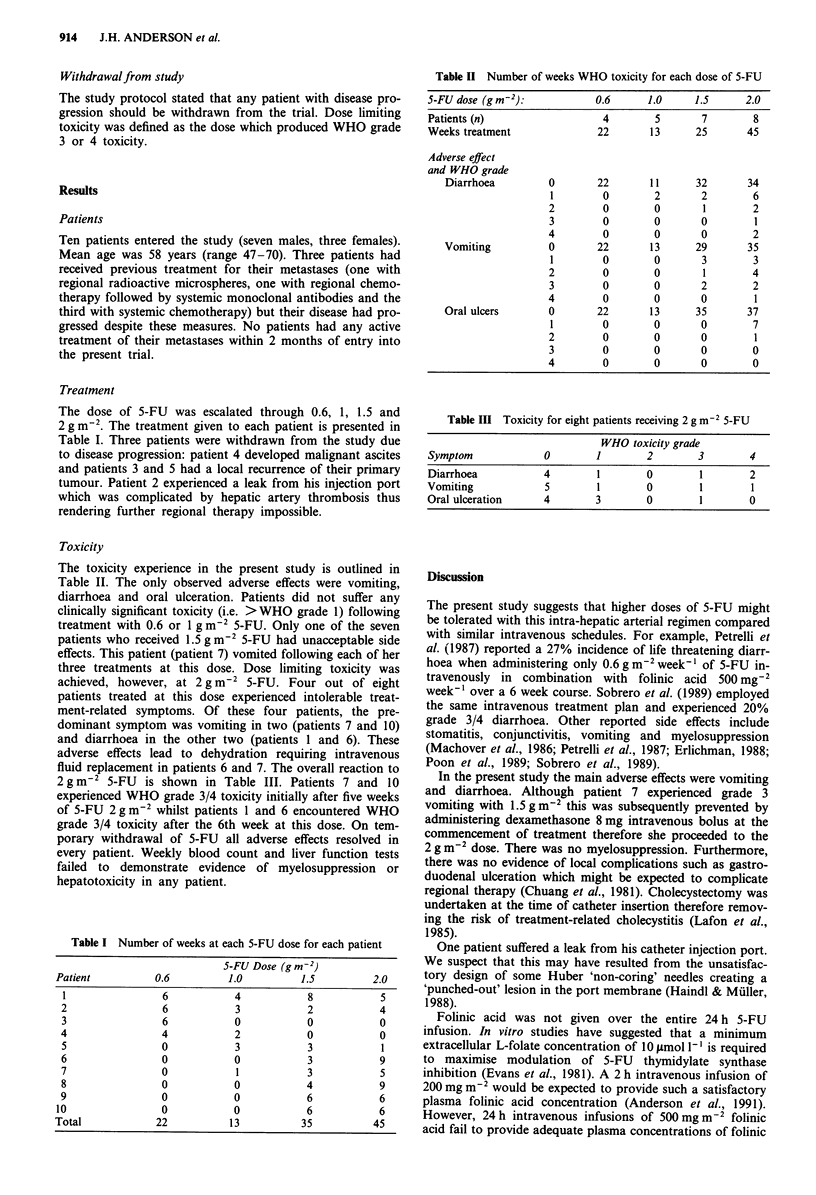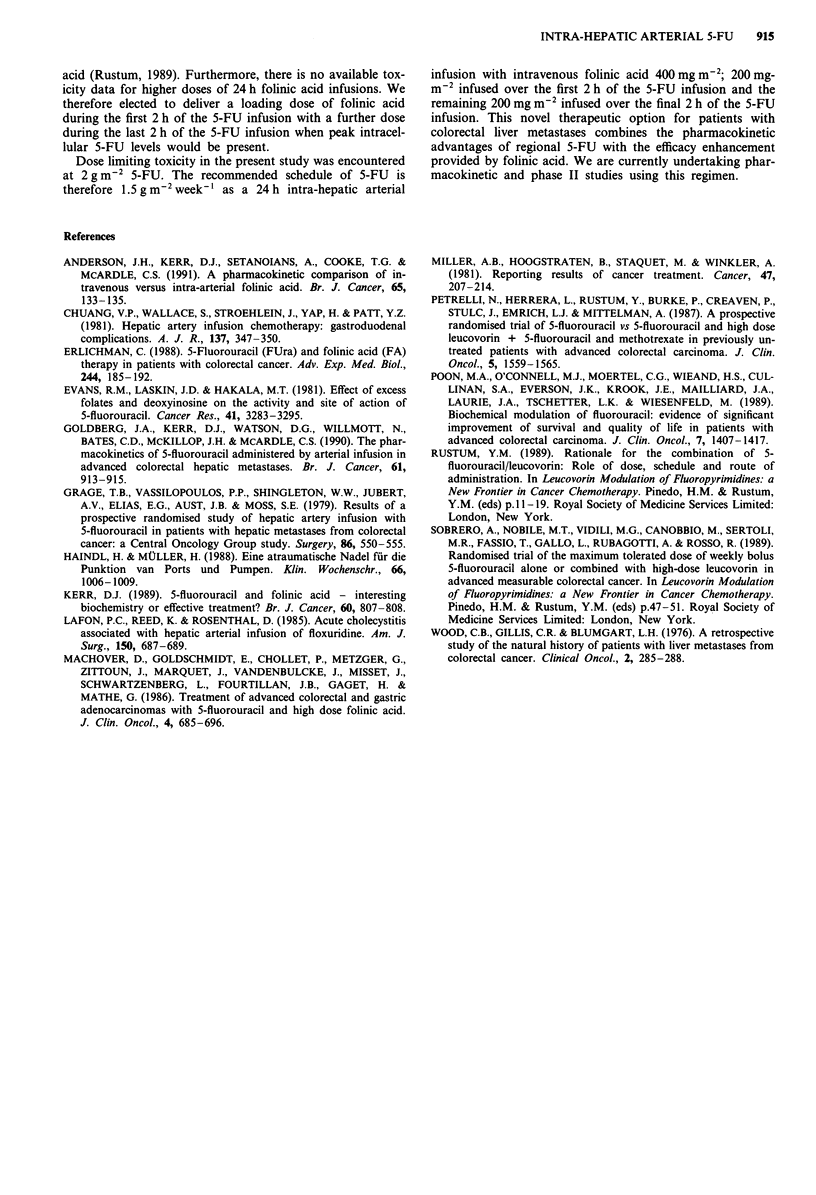# A phase I study of regional 5-fluorouracil and systemic folinic acid for patients with colorectal liver metastases.

**DOI:** 10.1038/bjc.1992.191

**Published:** 1992-06

**Authors:** J. H. Anderson, D. J. Kerr, T. G. Cooke, C. S. McArdle

**Affiliations:** University Department of Surgery, Royal Infirmary, Glasgow, UK.

## Abstract

A phase I study was undertaken in order to establish the maximum tolerated dose of intra-hepatic arterial 5-fluorouracil (5-FU) when given in combination with systemic folinic acid. Patients with colorectal liver metastases (n = 10) received escalating doses of 5-FU as a 24 h infusion with a fixed dose (400 mg m-2) of intravenous folinic acid once per week. Dose limiting toxicity (WHO grade greater than 2) was encountered at 2 g m-2 5-FU. Principal adverse effects were diarrhoea, vomiting and oral ulceration. The recommended dose for phase II studies is 1.5 g m-2 week-1 24 h 5-FU regional infusion with 400 mg m-2 week-1 intravenous folinic acid.


Br. J. Cancer (1992), 65, 913-915 © Macmillan Press Ltd., 1992

### A phase I study of regional 5-fluorouracil and systemic folinic acid for patients with colorectal liver metastases

J.H. Anderson[1], D.J. Kerr[2], T.G. Cooke[1] & C.S. McArdle[1]

[1]*University Department of Surgery, The Royal Infirmary, Glasgow, G31 2ER;* [2]*CRC Department of Medical Oncology, Glasgow University, Glasgow, G61 1BD, UK.*

**Summary** A phase I study was undertaken in order to establish the maximum tolerated dose of intra-hepatic arterial 5-fluorouracil (5-FU) when given in combination with systemic folinic acid. Patients with colorectal liver metastases ($n = 10$) received escalating doses of 5-FU as a 24 h infusion with a fixed dose (400 mg $m^{-2}$) of intravenous folinic acid once per week. Dose limiting toxicity (WHO grade $>2$) was encountered at 2 g $m^{-2}$ 5-FU. Principal adverse effects were diarrhoea, vomiting and oral ulceration. The recommended dose for phase II studies is 1.5 g $m^{-2}$ $week^{-1}$ 24 h 5-FU regional infusion with 400 mg $m^{-2}$ $week^{-1}$ intravenous folinic acid.

The prognosis for patients with colorectal liver metastases remains depressing; the mean survival in the West of Scotland is approximately 3 months (Wood *et al.*, 1976). The results of systemic chemotherapy have generally been regarded as disappointing. However, recent randomised trials have suggested that the addition of folinic acid may significantly improve survival amongst advanced colorectal cancer patients receiving intravenous 5-FU (Erlichman, 1988; Poon *et al.*, 1989; Kerr, 1989). Unfortunately, this advance has not been without toxicity, which varies according to drug dose and schedule.

An alternative therapeutic strategy is provided by regional treatment via a hepatic artery catheter. Higher drug concentrations are achieved in the liver with relatively less drug escaping into the systemic vascular compartment therefore diminishing systemic toxicity. We have shown in a series of previous pharmacokinetically guided studies that 24 h intra-arterial infusion of 5-FU confers a significant pharmacological advantage relative to intravenous infusions or intra-arterial bolus administration (Goldberg *et al.*, 1990). Regional administration of folinic acid is also associated with a statistically significant reduction in systemic exposure compared with the intravenous route but this apparent pharmacokinetic advantage has been offset by the potential for hepatic artery catheter thrombosis with this drug (Anderson *et al.*, 1991).

For patients with colorectal liver metastases, regional 5-FU increases response rates compared with intravenous treatment, but there is no convincing evidence that survival is significantly prolonged (Grage *et al.*, 1979). Disease generally progresses through development of extra-hepatic metastases. It would appear, therefore, that many patients with metastatic disease, which is apparently confined to the liver, have occult micrometastases at other sites.

Clinically, there are two strategies to try to overcome this problem of systemic relapse following loco-regional therapy: combination of regional with systemic therapy or dose escalating the regional therapy until peripheral venous concentrations equal those which are achieved with conventional systemic treatment. We have decided to adopt the latter approach.

The aim the present study was to establish the maximum tolerated dose of a 24 h intra-hepatic arterial infusion of 5-FU given in combination with a fixed dose of intravenous folinic acid. This might allow the generation of high 5-FU levels within the liver, the site of predominant bulk disease, whilst maintaining adequate systemic levels.

Correspondence: J.H. Anderson.
Received 13 August 1991; accepted 20 December 1992.

### Patients and methods

### *Patients*

Patients with biopsy-proven, metastatic, colorectal adenocarcinoma, confined to the liver were recruited. All subjects had previously undergone a resection of an adenocarcinoma of the colon or rectum and surgical placement of a hepatic artery catheter (Infusaport 38940. Shiley Infusaid Inc., Norwood, MA, USA). The gall-bladder had been removed and branches of the hepatic artery to extrahepatic organs were ligated. Following insertion of the catheter, it had been perfused with methylene blue to ensure that tissues outwith the liver were not subjected to the intra-arterial chemotherapy.

Entry criteria were; WHO performance status $<2$, life expectancy $>2$ months, white cell count $>4 \times 10^9 l^{-1}$, platelets $>150 \times 10^9 l^{-1}$ and bilirubin $<30 \mu mol l^{-1}$. Prior to commencing treatment, and during each 2-week break between courses, disease was staged using abdominal CT scan and chest radiograph. All patients gave informed consent before entering the study.

### *Treatment*

Patients were treated once per week. Each course of therapy lasted 6 weeks and there was a 2 week break between courses. 5-FU (25 mg $ml^{-1}$) was delivered percutaneously into the Infusaport arterial catheter as a continuous 24 h infusion. The starting dose of 5-FU was 600 mg $m^{-2}$ $week^{-1}$: similar to that recommended for intravenous use (Petrelli *et al.*, 1987). The dose of 5-FU was increased once three patients had received that dose for at least 3 consecutive weeks without unacceptable toxicity. Heparin (100 U $ml^{-1}$) was added to the infusion to reduce the risk of catheter thrombosis. Folinic acid was administered intravenously at a fixed total dose of 400 mg $m^{-2}$ $week^{-1}$; 200 mg $m^{-2}$ was infused over the first 2 h of the 5-FU infusion and the remaining 200 mg $m^{-2}$ was infused over the final 2 h of the 5-FU infusion.

### *Toxicity assessments*

Patients were assessed once per week and full blood count, liver function tests and serum urea and electrolytes were measured. Toxicity was assessed using standard WHO criteria on a scale from 0 (absent) to 4 (severe) for each adverse effect (Miller *et al.*, 1981). Particular attention was paid to oral ulceration, vomiting, diarrhoea, 'hand foot syndrome', alopecia, skin rashes, myelosuppression and serum bilirubin, AST, ALT, alkaline phosphatase, urea and creatinine.

*Withdrawal from study*

The study protocol stated that any patient with disease progression should be withdrawn from the trial. Dose limiting toxicity was defined as the dose which produced WHO grade 3 or 4 toxicity.

### Results

*Patients*

Ten patients entered the study (seven males, three females). Mean age was 58 years (range 47–70). Three patients had received previous treatment for their metastases (one with regional radioactive microspheres, one with regional chemotherapy followed by systemic monoclonal antibodies and the third with systemic chemotherapy) but their disease had progressed despite these measures. No patients had any active treatment of their metastases within 2 months of entry into the present trial.

*Treatment*

The dose of 5-FU was escalated through 0.6, 1, 1.5 and $2\,g\,m^{-2}$. The treatment given to each patient is presented in Table I. Three patients were withdrawn from the study due to disease progression: patient 4 developed malignant ascites and patients 3 and 5 had a local recurrence of their primary tumour. Patient 2 experienced a leak from his injection port which was complicated by hepatic artery thrombosis thus rendering further regional therapy impossible.

*Toxicity*

The toxicity experience in the present study is outlined in Table II. The only observed adverse effects were vomiting, diarrhoea and oral ulceration. Patients did not suffer any clinically significant toxicity (i.e. $>$WHO grade 1) following treatment with 0.6 or $1\,g\,m^{-2}$ 5-FU. Only one of the seven patients who received $1.5\,g\,m^{-2}$ 5-FU had unacceptable side effects. This patient (patient 7) vomited following each of her three treatments at this dose. Dose limiting toxicity was achieved, however, at $2\,g\,m^{-2}$ 5-FU. Four out of eight patients treated at this dose experienced intolerable treatment-related symptoms. Of these four patients, the predominant symptom was vomiting in two (patients 7 and 10) and diarrhoea in the other two (patients 1 and 6). These adverse effects lead to dehydration requiring intravenous fluid replacement in patients 6 and 7. The overall reaction to $2\,g\,m^{-2}$ 5-FU is shown in Table III. Patients 7 and 10 experienced WHO grade 3/4 toxicity initially after five weeks of 5-FU $2\,g\,m^{-2}$ whilst patients 1 and 6 encountered WHO grade 3/4 toxicity after the 6th week at this dose. On temporary withdrawal of 5-FU all adverse effects resolved in every patient. Weekly blood count and liver function tests failed to demonstrate evidence of myelosuppression or hepatotoxicity in any patient.

**Table I** Number of weeks at each 5-FU dose for each patient

| | 5-FU Dose ($g\,m^{-2}$) | | | |
|---|---|---|---|---|
| *Patient* | *0.6* | *1.0* | *1.5* | *2.0* |
| 1 | 6 | 4 | 8 | 5 |
| 2 | 6 | 3 | 2 | 4 |
| 3 | 6 | 0 | 0 | 0 |
| 4 | 4 | 2 | 0 | 0 |
| 5 | 0 | 3 | 3 | 1 |
| 6 | 0 | 0 | 3 | 9 |
| 7 | 0 | 1 | 3 | 5 |
| 8 | 0 | 0 | 4 | 9 |
| 9 | 0 | 0 | 6 | 6 |
| 10 | 0 | 0 | 6 | 6 |
| Total | 22 | 13 | 35 | 45 |

**Table II** Number of weeks WHO toxicity for each dose of 5-FU

| *5-FU dose ($g\,m^{-2}$):* | | *0.6* | *1.0* | *1.5* | *2.0* |
|---|---|---|---|---|---|
| Patients (*n*) | | 4 | 5 | 7 | 8 |
| Weeks treatment | | 22 | 13 | 25 | 45 |
| *Adverse effect and WHO grade* | | | | | |
| Diarrhoea | 0 | 22 | 11 | 32 | 34 |
| | 1 | 0 | 2 | 2 | 6 |
| | 2 | 0 | 0 | 1 | 2 |
| | 3 | 0 | 0 | 0 | 1 |
| | 4 | 0 | 0 | 0 | 2 |
| Vomiting | 0 | 22 | 13 | 29 | 35 |
| | 1 | 0 | 0 | 3 | 3 |
| | 2 | 0 | 0 | 1 | 4 |
| | 3 | 0 | 0 | 2 | 2 |
| | 4 | 0 | 0 | 0 | 1 |
| Oral ulcers | 0 | 22 | 13 | 35 | 37 |
| | 1 | 0 | 0 | 0 | 7 |
| | 2 | 0 | 0 | 0 | 1 |
| | 3 | 0 | 0 | 0 | 0 |
| | 4 | 0 | 0 | 0 | 0 |

**Table III** Toxicity for eight patients receiving $2\,g\,m^{-2}$ 5-FU

| | WHO toxicity grade | | | | |
|---|---|---|---|---|---|
| *Symptom* | *0* | *1* | *2* | *3* | *4* |
| Diarrhoea | 4 | 1 | 0 | 1 | 2 |
| Vomiting | 5 | 1 | 0 | 1 | 1 |
| Oral ulceration | 4 | 3 | 0 | 1 | 0 |

### Discussion

The present study suggests that higher doses of 5-FU might be tolerated with this intra-hepatic arterial regimen compared with similar intravenous schedules. For example, Petrelli *et al.* (1987) reported a 27% incidence of life threatening diarrhoea when administering only $0.6\,g\,m^{-2}\,week^{-1}$ of 5-FU intravenously in combination with folinic acid $500\,mg^{-2}\,week^{-1}$ over a 6 week course. Sobrero *et al.* (1989) employed the same intravenous treatment plan and experienced 20% grade 3/4 diarrhoea. Other reported side effects include stomatitis, conjunctivitis, vomiting and myelosuppression (Machover *et al.*, 1986; Petrelli *et al.*, 1987; Erlichman, 1988; Poon *et al.*, 1989; Sobrero *et al.*, 1989).

In the present study the main adverse effects were vomiting and diarrhoea. Although patient 7 experienced grade 3 vomiting with $1.5\,g\,m^{-2}$ this was subsequently prevented by administering dexamethasone 8 mg intravenous bolus at the commencement of treatment therefore she proceeded to the $2\,g\,m^{-2}$ dose. There was no myelosuppression. Furthermore, there was no evidence of local complications such as gastroduodenal ulceration which might be expected to complicate regional therapy (Chuang *et al.*, 1981). Cholecystectomy was undertaken at the time of catheter insertion therefore removing the risk of treatment-related cholecystitis (Lafon *et al.*, 1985).

One patient suffered a leak from his catheter injection port. We suspect that this may have resulted from the unsatisfactory design of some Huber 'non-coring' needles creating a 'punched-out' lesion in the port membrane (Haindl & Müller, 1988).

Folinic acid was not given over the entire 24 h 5-FU infusion. *In vitro* studies have suggested that a minimum extracellular L-folate concentration of $10\,\mu mol\,l^{-1}$ is required to maximise modulation of 5-FU thymidylate synthase inhibition (Evans *et al.*, 1981). A 2 h intravenous infusion of $200\,mg\,m^{-2}$ would be expected to provide such a satisfactory plasma folinic acid concentration (Anderson *et al.*, 1991). However, 24 h intravenous infusions of $500\,mg\,m^{-2}$ folinic acid fail to provide adequate plasma concentrations of folinic acid (Rustum, 1989). Furthermore, there is no available toxicity data for higher doses of 24 h folinic acid infusions. We therefore elected to deliver a loading dose of folinic acid during the first 2 h of the 5-FU infusion with a further dose during the last 2 h of the 5-FU infusion when peak intracellular 5-FU levels would be present.

Dose limiting toxicity in the present study was encountered at $2\,g\,m^{-2}$ 5-FU. The recommended schedule of 5-FU is therefore $1.5\,g\,m^{-2}\,week^{-1}$ as a 24 h intra-hepatic arterial infusion with intravenous folinic acid $400\,mg\,m^{-2}$; $200\,mg\,m^{-2}$ infused over the first 2 h of the 5-FU infusion and the remaining $200\,mg\,m^{-2}$ infused over the final 2 h of the 5-FU infusion. This novel therapeutic option for patients with colorectal liver metastases combines the pharmacokinetic advantages of regional 5-FU with the efficacy enhancement provided by folinic acid. We are currently undertaking pharmacokinetic and phase II studies using this regimen.

### References

ANDERSON, J.H., KERR, D.J., SETANOIANS, A., COOKE, T.G. & MCARDLE, C.S. (1991). A pharmacokinetic comparison of intravenous versus intra-arterial folinic acid. *Br. J. Cancer*, **65**, 133-135.

CHUANG, V.P., WALLACE, S., STROEHLEIN, J., YAP, H. & PATT, Y.Z. (1981). Hepatic artery infusion chemotherapy: gastroduodenal complications. *A. J. R.*, **137**, 347-350.

ERLICHMAN, C. (1988). 5-Fluorouracil (FUra) and folinic acid (FA) therapy in patients with colorectal cancer. *Adv. Exp. Med. Biol.*, **244**, 185-192.

EVANS, R.M., LASKIN, J.D. & HAKALA, M.T. (1981). Effect of excess folates and deoxyinosine on the activity and site of action of 5-fluorouracil. *Cancer Res.*, **41**, 3283-3295.

GOLDBERG, J.A., KERR, D.J., WATSON, D.G., WILLMOTT, N., BATES, C.D., MCKILLOP, J.H. & MCARDLE, C.S. (1990). The pharmacokinetics of 5-fluorouracil administered by arterial infusion in advanced colorectal hepatic metastases. *Br. J. Cancer*, **61**, 913-915.

GRAGE, T.B., VASSILOPOULOS, P.P., SHINGLETON, W.W., JUBERT, A.V., ELIAS, E.G., AUST, J.B. & MOSS, S.E. (1979). Results of a prospective randomised study of hepatic artery infusion with 5-fluorouracil in patients with hepatic metastases from colorectal cancer: a Central Oncology Group study. *Surgery*, **86**, 550-555.

HAINDL, H. & MÜLLER, H. (1988). Eine atraumatische Nadel für die Punktion van Ports und Pumpen. *Klin. Wochenschr.*, **66**, 1006-1009.

KERR, D.J. (1989). 5-fluorouracil and folinic acid – interesting biochemistry or effective treatment? *Br. J. Cancer*, **60**, 807-808.

LAFON, P.C., REED, K. & ROSENTHAL, D. (1985). Acute cholecystitis associated with hepatic arterial infusion of floxuridine. *Am. J. Surg.*, **150**, 687-689.

MACHOVER, D., GOLDSCHMIDT, E., CHOLLET, P., METZGER, G., ZITTOUN, J., MARQUET, J., VANDENBULCKE, J., MISSET, J., SCHWARTZENBERG, L., FOURTILLAN, J.B., GAGET, H. & MATHE, G. (1986). Treatment of advanced colorectal and gastric adenocarcinomas with 5-fluorouracil and high dose folinic acid. *J. Clin. Oncol.*, **4**, 685-696.

MILLER, A.B., HOOGSTRATEN, B., STAQUET, M. & WINKLER, A. (1981). Reporting results of cancer treatment. *Cancer*, **47**, 207-214.

PETRELLI, N., HERRERA, L., RUSTUM, Y., BURKE, P., CREAVEN, P., STULC, J., EMRICH, L.J. & MITTELMAN, A. (1987). A prospective randomised trial of 5-fluorouracil *vs* 5-fluorouracil and high dose leucovorin + 5-fluorouracil and methotrexate in previously untreated patients with advanced colorectal carcinoma. *J. Clin. Oncol.*, **5**, 1559-1565.

POON, M.A., O'CONNELL, M.J., MOERTEL, C.G., WIEAND, H.S., CULLINAN, S.A., EVERSON, J.K., KROOK, J.E., MAILLIARD, J.A., LAURIE, J.A., TSCHETTER, L.K. & WIESENFELD, M. (1989). Biochemical modulation of fluorouracil: evidence of significant improvement of survival and quality of life in patients with advanced colorectal carcinoma. *J. Clin. Oncol.*, **7**, 1407-1417.

RUSTUM, Y.M. (1989). Rationale for the combination of 5-fluorouracil/leucovorin: Role of dose, schedule and route of administration. In *Leucovorin Modulation of Fluoropyrimidines: a New Frontier in Cancer Chemotherapy*. Pinedo, H.M. & Rustum, Y.M. (eds) p.11-19. Royal Society of Medicine Services Limited: London, New York.

SOBRERO, A., NOBILE, M.T., VIDILI, M.G., CANOBBIO, M., SERTOLI, M.R., FASSIO, T., GALLO, L., RUBAGOTTI, A. & ROSSO, R. (1989). Randomised trial of the maximum tolerated dose of weekly bolus 5-fluorouracil alone or combined with high-dose leucovorin in advanced measurable colorectal cancer. In *Leucovorin Modulation of Fluoropyrimidines: a New Frontier in Cancer Chemotherapy*. Pinedo, H.M. & Rustum, Y.M. (eds) p.47-51. Royal Society of Medicine Services Limited: London, New York.

WOOD, C.B., GILLIS, C.R. & BLUMGART, L.H. (1976). A retrospective study of the natural history of patients with liver metastases from colorectal cancer. *Clinical Oncol.*, **2**, 285-288.